# Abscopal Effect in Radiation Therapy: The Immune Mechanisms and Clinical Advances

**DOI:** 10.1002/mco2.70932

**Published:** 2026-08-17

**Authors:** Yihong Dong, Moran Chen, Xupeng Hou, Xiaoya Tang, Xiaoli Yu, Jing Liu

**Affiliations:** ^1^ Key Laboratory of Breast Cancer in Shanghai Department of Breast Surgery Fudan University Shanghai Cancer Center Shanghai China; ^2^ Department of Oncology Shanghai Medical College Fudan University Shanghai China; ^3^ Department of Pancreatic Cancer Tianjin Medical University Cancer Institute and Hospital National Clinical Research Center for Cancer Key Laboratory of Cancer Prevention and Therapy Tianjin's Clinical Research Center for Cancer Tianjin China; ^4^ Department of Radiation Oncology Fudan University Shanghai Cancer Center Shanghai China; ^5^ Shanghai Clinical Research Center for Radiation Oncology Shanghai Key Laboratory of Radiation Oncology Shanghai China

**Keywords:** abscopal effect, radioimmunotherapy, radiotherapy

## Abstract

The abscopal effect is a systemic antitumor response to localized radiotherapy (RT) that has emerged as a central focus in the era of immunotherapy. Clinical evidence demonstrates that local RT can induce regression of distant, nonirradiated metastases, including cases of complete remission. However, the effect remains unpredictable, with marked variation in clinical outcomes ranging from complete regression to paradoxical progression. The mechanistic basis for this heterogeneity and optimal strategies to enhance abscopal effects remain incompletely understood. This review synthesizes current evidence into a mechanistic framework organized around a three‐stage immune cascade: ignition, orchestration, and execution. We examine how tumor‐intrinsic factors, RT parameters, and biomarkers shape this cascade. We then appraise the clinical trial landscape and discuss how these mechanistic insights may inform patient selection, treatment timing, and radioimmunotherapy combination strategies. Critically, we establish a bidirectional framework distinguishing the favorable abscopal response from the pro‐tumorigenic badscopal effect, in which RT paradoxically promotes distant progression through immunosuppressive pathways. By clarifying these mechanisms and variables, this review offers a framework for improving future clinical trial design and advancing the abscopal effect from a sporadically observed phenomenon to a predictable therapeutic strategy.

## Graphical abstract

The schematic outlines the key events of the abscopal effect. (Ignition) Local radiotherapy (RT) induces the release of immunogenic signals from the tumor, leading to dendritic cell (DC) activation. (Orchestration) In the tumor‐draining lymph node (TDLN), DCs prime naive T cells into cytotoxic T lymphocytes (CTLs), a process modulated by regulatory T cells (Tregs). (Execution) Activated CTLs traffic to distant tumors to kill cancer cells, encountering resistance from the immunosuppressive microenvironment.

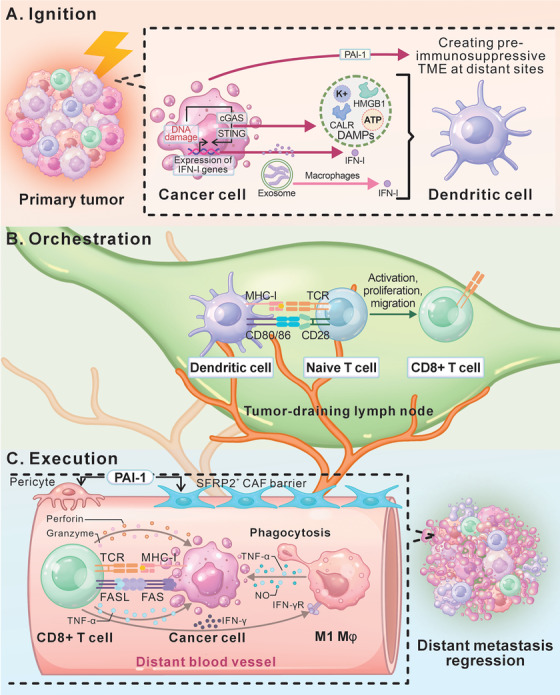

## 1 Introduction

Radiotherapy (RT) is recommended for most solid tumors, and can improve survival in over a quarter of patients [1, 2]. Conventionally, RT is used as a loco‐regional treatment, although metastasis‐directed approaches may benefit selected oligometastatic settings; spontaneous distant regression after local RT remains uncommon [3, 4]. However, accumulating evidence shows that RT can also elicit systemic immune responses [5].

**FIGURE 1:**
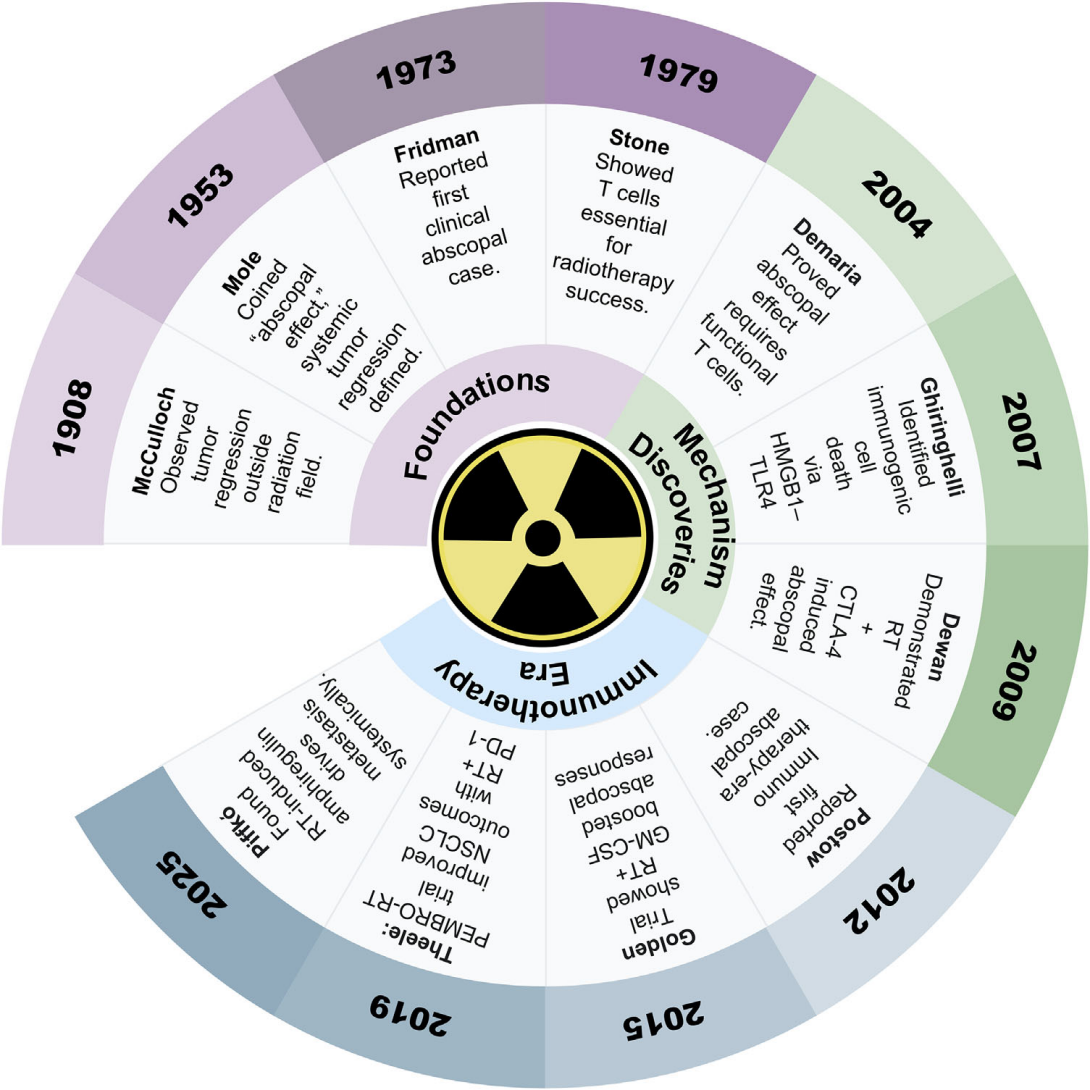
Key milestones in the history of the abscopal effect. Timeline of major discoveries from initial observations to clinical translation, organized into three eras: foundations (1908–1979), establishing T cell dependency; mechanism discoveries (2004–2009), identifying immunogenic cell death pathways; and immunotherapy era (2012–present), demonstrating radio‐immunotherapy synergy.

Among these systemic immune responses [6], the abscopal effect describes regression of nonirradiated distant lesions following localized RT [7, 8]. R.H. Mole introduced it in 1953 after observing regression of shielded tumors in animal models [9]. Figure 1 summarizes key historical milestones from 1908 to the present.

Early abscopal reports remained rare for decades [10]. This changed with immune checkpoint inhibitors (ICIs) targeting cytotoxic T‐lymphocyte‐associated protein 4 (CTLA‐4) and programmed cell death protein 1 and programmed cell death ligand 1 (PD‐1 and PD‐L1), which were associated with increased reports of abscopal responses when combined with RT. RT is increasingly viewed as a treatment that can shape antitumor immunity beyond the irradiated field.

Despite these advances, abscopal effects remain unpredictable and inconsistent. Some patients achieve systemic regression, while others show no response or even out‐of‐field progression [11]. The determinants of this variability are poorly understood. Tumor heterogeneity, inconsistent trial standards, and uncertainty about optimal RT parameters all contribute to this knowledge gap. A comprehensive synthesis of existing evidence is therefore needed.

This review first summarizes the immune mechanisms of the abscopal effect as a three‐stage cascade of ignition, orchestration, and execution. It then organizes the main determinants of response into tumor type heterogeneity, radiation therapy parameters, and biomarkers for prediction and monitoring. Subsequent sections review the clinical trial landscape, discuss practical optimization of radioimmunotherapy synergy, and consider the badscopal phenomenon together with unresolved questions for future research.

## 2 Mechanisms of the Abscopal Effect

**FIGURE 2:**
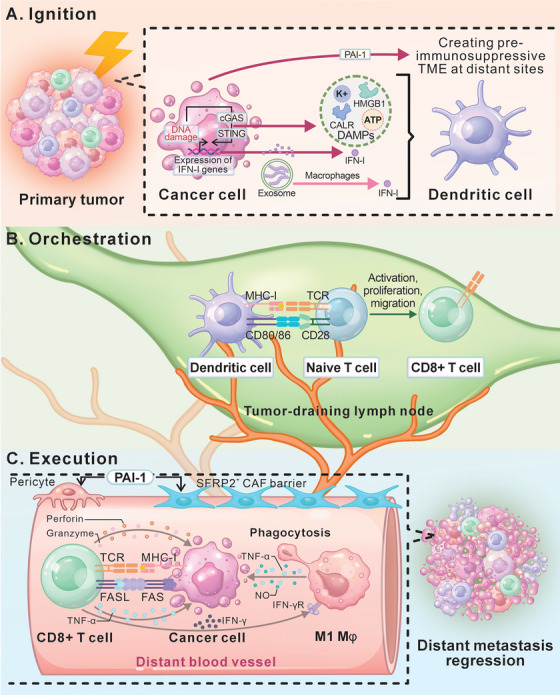
Three‐stage mechanism of RT‐induced abscopal effect. Ignition: RT induces immunogenic signals and DC activation. Orchestration: DCs prime naive T cells into CTLs in tumor‐draining lymph nodes, modulated by Tregs. Execution: CTLs traffic to distant tumors, overcoming immunosuppressive resistance.

The abscopal effect can be understood through the cancer‐immunity cycle framework of Chen and Mellman [12]. RT converts a tumor into an in situ vaccine, initiating a systemic T cell response [13]. We frame it as three stages: ignition, orchestration, and execution [6] (Figure 2).

### 2.1 Ignition: Radiation‐Induced Immunogenic Cell Death (ICD)

The cascade begins when irradiated tumor cells undergo ICD. This releases damage‐associated molecular patterns (DAMPs), including calreticulin [14], HMGB1 [15], and ATP [16]. These molecules act as signals that recruit antigen‐presenting cells [17, 18, 19].

At the same time, DNA from dying tumor cells escapes into the cytosol, activating the cyclic GMP‐AMP synthase (cGAS)–stimulator of interferon genes (STING) pathway and inducing Type I interferon (IFN‐I) production, which is further amplified by tumor‐derived exosomes [20, 21, 22]. IFN‐I signaling is central to the abscopal effect [19, 23, 24], as it is crucial for activating dendritic cells (DCs) and priming antitumor T cells [25].

However, the ignition phase is subject to multiple regulatory constraints. The radiation regimen itself is an important variable. For instance, high single doses may induce the exonuclease TREX1, which degrades cytosolic DNA and weakens STING signaling [26]. In addition to external factors, tumor cells can intrinsically weaken this initial signal through adaptive resistance, such as inducing cholesterol biosynthesis to sequester STING or by leveraging p53 mutations to impair STING signaling [27, 28]. Furthermore, RT can trigger systemic suppressive signals. Irradiated tumors release plasminogen activator inhibitor‐1 (PAI‐1), which travels through the bloodstream to remotely establish an immunosuppressive microenvironment at distant tumor sites, thereby blunting the efficacy of infiltrating immune cells [29]. Therefore, the ultimate strength of the ignition phase depends on the balance between these pro‐activating and suppressive signals.

### 2.2 Orchestration: Antigen Presentation and T Cell Priming

After ignition, IFN‐I signaling drives DC maturation and migration to tumor‐draining lymph nodes (TDLNs) [22, 30]. There, antigen‐loaded DCs prime naïve T cells, expanding tumor‐specific CD8^+^ cytotoxic T lymphocytes (CTLs) [31, 32]. This priming is counteracted by regulatory T cells (Tregs) [33, 34] and immunomodulatory cytokines such as transforming growth factor beta (TGF‐β) [30, 35, 36], which suppress antigen presentation and T cell activation. The overall magnitude of the immune response is therefore determined by the balance between activation and suppression within lymphoid organs.

Beyond TDLNs, tertiary lymphoid structures (TLS) represent an additional site of adaptive immune orchestration. These ectopic lymphoid aggregates form in chronically inflamed nonlymphoid tissues and serve as local hubs for antigen presentation, B cell activation, and effector T cell priming. High‐density mature TLS within solid tumors has been independently associated with improved responses to ICIs across multiple cancer lineages [37, 38]. RT‐induced inflammation may promote de novo TLS formation, potentially sustaining immune priming beyond the acute irradiation window [38]. TLS density and maturation status may therefore indicate whether orchestration‐phase immunity is preserved, although their clinical utility requires dedicated validation.

### 2.3 Execution: Systemic T Cell Trafficking and Tumor Killing

Once primed, CD8^+^ T cells circulate through the bloodstream to target distant, nonirradiated tumor sites. A sustained T cell response is required for this execution phase. This killing phase is mediated mainly by CTLs [39]. Specifically, systemic IFN‐I provides the “final arming” by boosting their secretion of molecules such as granzyme B, which directly lyses tumor cells, and interferon‐gamma (IFN‐γ), which improves immune recognition [19]. As detailed in Section 3.3, IFN‐γ levels have been reported as a systemic correlate of abscopal responses [40]. Recent evidence challenges the traditional CTL‐centric view. A synergistic licensing‐execution model has been proposed [41]. While CD8^+^ T cells mediate antigen‐specific recognition and provide immune licensing signals, activated tumor‐associated macrophages (TAMs) often serve as major execution‐phase effector cells. They eliminate tumor cells via phagocytosis [42]. Thus, an effective abscopal effect relies on this complementary partnership: T cells provide the licensing signal, while TAMs execute tumor cell elimination.

In the distant tumor microenvironment (TME), infiltrating immune cells encounter stromal and myeloid barriers. PAI‐1‐ and SFRP2‐positive cancer‐associated fibroblasts can limit T cell infiltration, whereas PD‐L1 expression, myeloid‐derived suppressor cells (MDSCs), and M2 TAMs suppress effector function [43, 44]. Strategies that remodel hypoxia, metabolism, or nitric oxide signaling have shown preclinical potential to restore T cell activity at distant sites [45, 46, 47]. These pathways are relevant to execution‐phase remodeling [48]. Tumor‐derived exosomes may also shape the execution‐phase microenvironment [49].

The abscopal effect therefore depends on whether radiation‐induced tumor damage is converted into systemic immune execution. The following sections examine how tumor context, RT design, biomarkers, and therapeutic combinations shape this process.

## 3 Key Determinants of Abscopal Response

### 3.1 Tumor Type Heterogeneity

Tumor lineage and immune contexture are major determinants because each malignancy differs in whether it can complete the ignition, orchestration, and execution stages of the abscopal cascade. The distribution of reported clinical cases shows both recurrent patterns and important reporting biases.

**FIGURE 3:**
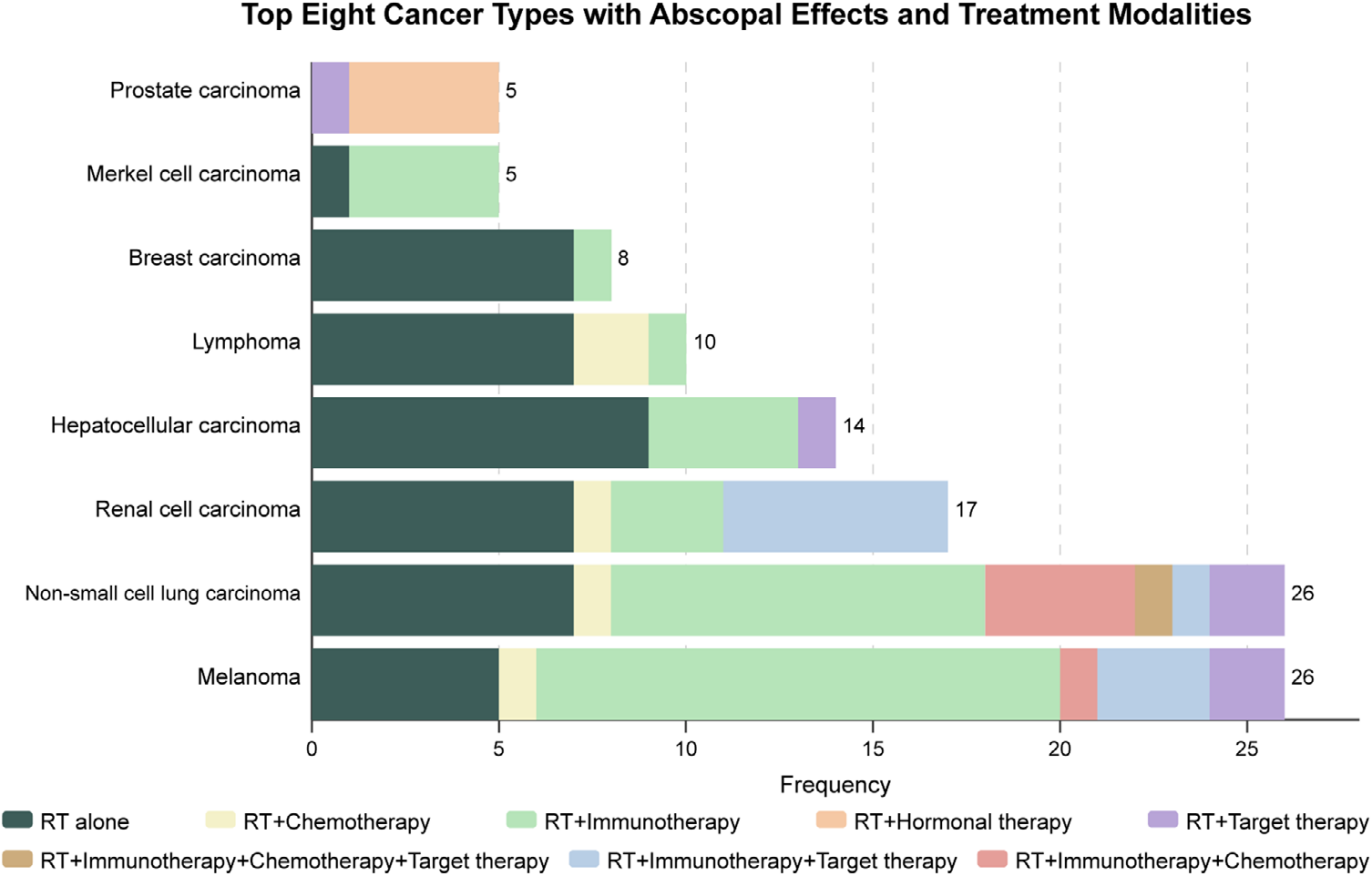
Top eight cancer types with abscopal effects and treatment modalities. Frequency of reported abscopal effects across eight cancer types, stratified by treatment modality (RT alone or combined with chemotherapy, immunotherapy, or targeted therapy). Melanoma shows the highest frequency, predominantly with RT plus immunotherapy. *Note*: Reported frequency reflects trial volume and publication bias, not biological incidence.

Historical reports, together with our analysis of 177 reported cases, consistently show marked variability in the reported frequency of abscopal effects across cancer types [35, 50, 51] (Table S1). Melanoma (*n* = 26), non‐small cell lung cancer (NSCLC) (*n* = 26), renal cell carcinoma (RCC) (*n* = 17), hepatocellular carcinoma (*n* = 14), and lymphoma (*n* = 10) together represented more than half of the reported cases. This concentration likely reflects a bias stemming from the high volume of immunotherapy trials in melanoma and NSCLC [52]. Figure 3 thus represents the landscape of reported evidence rather than biological incidence.

At the same time, the phenomenon has been reported in more than 40 tumor types, ranging from breast cancer [53] and prostate cancer [54] to rare sarcomas [55], neuroendocrine tumors [56, 57], and even pheochromocytoma [58]. This highlights its occurrence across diverse tumor lineages, albeit predominantly in the form of sporadic case reports. However, reports remain sporadic in breast cancer, pancreatic ductal adenocarcinoma (PDAC) [59], and glioblastoma multiforme (GBM) [60]. The rarity of reported abscopal effects in these tumors correlates with biomarker signatures of immune exclusion, including high TGF‐β and absent T‐cell receptor (TCR) clonal expansion [61].

Ultimately, this scarcity is determined by the intrinsic immunological properties of the tumor and its metastatic niche. Immunogenicity is a major factor influencing the likelihood of an abscopal effect. Tumors with high mutational burden, abundant neoantigens, and strong capacity to activate effector T cells, such as melanoma [62, 63, 64], are more likely to elicit systemic responses. By contrast, tumors dominated by immunosuppressive cells and mediators tend to generate tolerance‐oriented microenvironments.

For hormone receptor‐positive breast cancers [65], bone metastasis creates a specific barrier. The bone marrow niche is enriched with TGF‐β, which actively excludes T cells and enforces an immunosuppressive state. Similarly, PDAC is enriched with MDSCs, Tregs, and dense stroma [66], creating formidable barriers at the execution stage, while GBM combines central nervous system immune privilege with glioma stem cell resistance to establish an almost impenetrable “immune desert” [67], similarly failing at the execution stage.

Functional T cell state and myeloid‐to‐lymphoid balance, not absolute counts, are decisive [68]. Thus, the challenge of inducing abscopal effects in these refractory tumors lies not only in activating antitumor immunity but also in dismantling deeply entrenched immunosuppressive programs.

Mechanistic differences in innate sensing also matter. RT can induce ICD and activate innate immune‐sensing pathways, including cGAS–STING, thereby promoting IFN‐I signaling [69]. However, IFN‐I is not uniformly beneficial: Acute signaling supports immune activation, whereas chronic or sustained signaling can reinforce immunosuppression and promote radioresistance [70]. Recent studies show that in PDAC, the IFN‐I response triggered by RT combined with ataxia telangiectasia mutated (ATM) inhibition is not dependent on cGAS–STING. Instead, it is mediated through the POLIII–RIG‐I–MAVS pathway [71]. This altered immune‐sensing mechanism suggests that distinct tumors may rely on different innate immune‐sensing pathways. Consequently, in immune “cold” tumors, this cascade often collapses early: Initial signals are inadequate, T cells remain suppressed, and TAMs cannot undergo polarization to execute the attack, ultimately halting the entire process.

In summary, the occurrence of abscopal effects can be understood as a sequence of checkpoints: (i) RT‐induced damage‐associated signals must be effectively recognized and amplified by the immune system [72, 73]; (ii) the regulatory direction of IFN‐I signaling determines whether immunity is promoted or suppressed [70]; and (iii) the functional synergy of T cells and myeloid cells within the distant TME dictates whether lesions can be eliminated [42]. Immune “hot” tumors often progress through these checkpoints, whereas “cold” tumors frequently falter. This heterogeneity highlights an important challenge: Tumor biology must be considered alongside the design of the radiation trigger itself, including dose, fractionation, field size, and modality.

### 3.2 Radiation Therapy Parameters as Modulators

Alongside tumor biology, the radiation trigger itself is a key determinant of whether local treatment generates systemic immunity. Abscopal effects have been observed across diverse RT modalities, including photon‐based approaches, brachytherapy, and radionuclide irradiation [54, 74, 75]. Particle therapies may also be relevant. Carbon ions, characterized by high linear energy transfer (LET), enhance tumor immunogenicity by intensifying DNA damage and activating the cGAS–STING pathway [76]. In contrast, proton therapy exploits the Bragg peak to spare surrounding immune organs, thereby preserving systemic immunity [77]. Clinical case reports have documented abscopal effects following particle RT in several cancer types, highlighting the potential of these advanced modalities [78].

The physical parameters of RT, especially dose and fractionation, exert a significant impact on the TME. Evidence supports a putative immunogenic window. Low‐dose RT (< 2 Gy) may function as an immunological adjuvant by remodeling the TME to increase responsiveness to immunotherapy [79, 80]. Mechanistically, these ultra‐low doses may enhance vascular permeability, promote TAM polarization, and stimulate pro‐inflammatory cytokine secretion, thereby facilitating synergy with immunotherapy [81].

Conversely, high‐dose RT represents a double‐edged sword. Although it promotes the release of antigens and DAMPs that stimulate immunity [82], it may also generate a suppressive microenvironment through vascular injury and MDSC recruitment [83]. In addition, high‐dose RT can induce hypoxia and upregulate hypoxia‐inducible factor 1‐alpha (HIF‐1α), which in turn recruits MDSCs and promotes immune cell apoptosis, thereby reinforcing an immunosuppressive milieu [84]. High doses may also upregulate the exonuclease TREX1, which diminishes the cGAS–STING response, offering a mechanistic explanation for this dose dependency [85]. Critically, TREX1 activation occurs predominantly at single doses exceeding 10–12 Gy per fraction, thereby supporting a putative immunogenic window for moderate hypofractionated regimens (8–12 Gy per fraction): These doses remain below the TREX1 threshold while being sufficient to induce ICD and Type I IFN signaling [26, 86]. Fractionation (e.g., 3 × 8 Gy or 5 × 6 Gy) may leverage this window and has shown greater efficacy than single large doses by maintaining cGAS–STING activation while avoiding TREX1‐mediated suppression, thereby balancing local tumor control with systemic immune activation [87]. This concept is supported by clinical data, including a retrospective study in which stereotactic body RT (SBRT) alone induced abscopal regression in approximately 25% of breast cancer patients [88]. In addition, biomarker‐guided dose selection, including tailoring fractionation to baseline tumor hypoxia and vascular perfusion, represents a potential personalization strategy.

Beyond radiation dose, the design of the irradiation field also influences the abscopal effect. Several studies indicate that partial tumor irradiation (PTI) can trigger immune responses. In certain contexts, PTI outperforms whole‐tumor irradiation. For example, a prospective clinical trial showed that PTI in patients with bulky tumors was associated with a high incidence of abscopal effects (41%) and durable survival benefits [89]. Preclinical evidence reinforces this immune dependence: Hemi‐tumor irradiation elicited abscopal effects in immunocompetent mice but failed to do so in T cell‐deficient nude mice [90]. These findings have given rise to the “radscopal effect” hypothesis, which proposes that creating spatial dose gradients within a tumor may optimize the priming of complementary immune pathways [91].

The efficacy of PTI is closely linked to the immunological context. In responsive models, the TME is enriched with effector memory T cells, whereas nonresponders are dominated by MDSCs [92]. To translate these findings into clinical practice, candidate dosimetric parameters have been proposed: Optimal PTI volumes range from 50% [90] to 70% [89], with 10 Gy per fraction to the targeted region. Jagodinsky et al. (2024) further demonstrated that intratumoral dose heterogeneity spanning 2–30 Gy maximizes abscopal efficacy when combined with checkpoint inhibition [91]. For clinical implementation, FDG‐PET (positron emission tomography)/CT‐guided delineation to selectively target hypovascular tumor cores while sparing the peritumoral immune microenvironment represents a rational approach. However, a prospective comparison of volume thresholds remains needed.

The immune benefit of field‐size reduction extends beyond the TME. Larger planning target volumes—particularly those encompassing central lymph node basins, major blood vessels, and the spleen—are independent dosimetric predictors of severe radiation‐induced lymphopenia [93, 94]. Notably, modeling evidence indicates that irradiation volume is a stronger driver of lymphocyte depletion than dose level itself at any fixed field size. Thus, restricting field extent carries immune‐protective value independent of dose prescription changes [95]. Reducing integral body dose thereby serves a dual purpose: It both preserves the peritumoral immune microenvironment exploited by PTI, and protects the circulating lymphocyte pool required for systemic immune execution at distant sites.

Prospective evidence supports this principle. A randomized trial comparing proton beam therapy with intensity‐modulated RT for esophageal cancer showed that the lower integral dose achieved by proton therapy translated into significantly higher absolute lymphocyte counts at the end of treatment [96]. This provides evidence that dosimetric choices in field design carry measurable systemic immune consequences.

Taken together, RT parameters influence the likelihood of abscopal effects and the immune context in which they arise. Because these effects vary with tumor context, biomarker‐based prediction and monitoring are needed to translate parameter optimization into patient‐level decision‐making.

### 3.3 Biomarkers for Predicting and Monitoring

Because tumor biology and RT design jointly determine whether the immune cascade can be completed, biomarkers are needed to identify responsive patients and guide dynamic treatment adaptation.

Among tumor‐intrinsic determinants, IFN signaling competence is paramount. Genetic ablation of IFN pathways abrogates abscopal effects. Conversely, elevated serum IFN‐γ following RT and ipilimumab predicts systemic responses in metastatic NSCLC [40].

Tumor‐intrinsic negative regulators also shape abscopal potential. Poly(ADP‐ribose) polymerase 7 (PARP7) has been reported in a Research Square preprint to suppress radiation‐induced interferon‐stimulated gene expression and necroptosis. PARP7 inhibition converted abscopal‐resistant tumors into responders in that preclinical setting [97], therefore, PARP7 should be regarded as a candidate negative predictive biomarker rather than a validated clinical marker. Baseline tumor immunogenicity reflected by neoantigen burden, MHC Class I presentation, and CD8^+^ T cell infiltration strongly predicts abscopal competence. Highly immunogenic models exhibit robust distant immune remodeling [98].

Within nonirradiated lesions, the balance between effector and suppressor populations determines outcome. Abscopal responders consistently display increased CD8^+^‐to‐Treg ratios and enhanced granzyme B expression [99]. Resistance is associated with Tregs and MDSC accumulation. Van der Woude et al. (2022) provided direct human evidence showing pembrolizumab plus SBRT significantly increased lymphocyte infiltration in nonirradiated NSCLC lesions compared to pembrolizumab alone [100].

Since routine biopsies of nonirradiated lesions are impractical, surrogate markers are critical. The CXCR3–C‐X‐C motif chemokine ligand 10 (CXCL10) chemokine axis is functionally essential. CXCR3 knockout abrogated abscopal effects despite intact T cell priming [99]. Intercellular adhesion molecule 1 (ICAM‐1) upregulation following RT is both necessary and sufficient for abscopal effects in preclinical models. ICAM‐1‐targeted PET imaging, performed early after RT, accurately predicted abscopal efficacy in murine models [101]. However, clinical validation remains ongoing.

TCR repertoire analysis can serve as a systemic monitor. Early dynamic changes in blood T cell clones have been strongly linked to systemic responses [40, 102]. Huang et al. (2025) revealed that clonal expansion of both preexisting and new tumor‐reactive T cells occurs in blood and nonirradiated sites, with neoantigen‐specific responses correlating with long‐term survival [103]. Moreover, studies on NBTXR3 nanoparticles confirm that RT‐enhanced immunogenicity drives measurable TCR diversity changes in distant tumors, supporting TCR dynamics as a readout of systemic priming [104].

Complementing T cell‐centric readouts, circulating tumor DNA (ctDNA) has emerged as an orthogonal systemic biomarker. It reflects tumor‐side dynamics rather than immune‐side priming. Each RT fraction causes a burst of acute tumor cell death. This produces a transient early elevation in ctDNA levels in some patients within days of starting treatment, followed by a sustained decline that tracks tumor debulking [105, 106]. The kinetics of this decline carry prognostic weight independent of conventional imaging. In a large prospective cohort of locally advanced NSCLC patients undergoing chemoradiotherapy, dynamic on‐treatment ctDNA trajectories predicted progression‐free survival (PFS) and overall survival (OS) significantly earlier than radiographic assessment. This identified high‐risk patients before clinically overt failure [107]. When chemoradiotherapy is followed by consolidation immunotherapy, serial ctDNA monitoring extends this utility further. Patients who remained ctDNA‐positive after completing RT derived the greatest survival benefit from subsequent immune checkpoint blockade. ctDNA‐negative patients maintained favorable outcomes regardless, enabling individualized de‐escalation decisions [108]. Integrated with TCR clonotype expansion and IFN‐γ trajectories, serial ctDNA monitoring offers real‐time assessment of whether a patient is on an abscopal or badscopal trajectory, potentially providing molecular feedback before imaging‐detectable changes occur.

TGF‐β is also a negative regulator. TGF‐β blockade markedly enhanced abscopal effects [61], positioning TGF‐β as both a resistance biomarker and therapeutic target. Functional imaging may provide further context: Preclinical and early translational data, including a bioRxiv preprint, suggest that pretreatment perfusion and hypoxia levels in irradiated lesions are associated with distant control [109, 110], as well‐perfused, normoxic tumors facilitate efficient DC migration and T cell trafficking.

As summarized in Table 1, abscopal effects depend on stage‐specific biomarkers. These markers identify patients who possess the necessary biological substrate. With such biomarkers defining patient selection criteria, the next critical step is examining how these principles translate into clinical practice through prospective trials.

**TABLE 1:** Core predictive biomarkers for abscopal effect.

Biomarker	Method	Impact	Mechanism and clinical implication
**I. Ignition phase**			
Tumor IFN‐I signature	Tumor RNA‐seq or IHC (pre‐RT)	Positive (+)	Required for DC activation; inhibited by PARP7 [40, 111]
PARP7 expression	Tumor IHC (baseline)	Negative (−)	Suppresses cytosolic DNA sensing and necroptosis [97]
Tumor immunogenicity (TMB and neoantigens)	WES or multiplex IHC	Positive (+)	Provides antigen targets for T cell recognition [98, 99]
Radiomics features	Baseline CT or MRI	Composite	Quantitative imaging features predict responder status noninvasively [109, 112]
Hypoxia and perfusion	DCE‐MRI or oximetry	Positive (+)	Normoxia supports ROS generation and T cell entry [109]
**II. Orchestration phase**			
Serum IFN‐γ	ELISA (Week 1 post‐RT)	Positive (+)	Indicates systemic immune activation [40]
TCR clonal dynamics	Blood TCR‐seq (serial)	Positive (+)	Tracks neoantigen‐specific priming [103, 104]
ctDNA clearance	Plasma cfDNA (serial)	Positive (+)	Early clearance surrogates distant lesion control [113, 114]
**III. Execution phase**			
CD8^+^‐to‐Treg ratio	Distant biopsy (post‐RT)	Positive (+)	High ratio correlates with tumor regression [99, 115]
ICAM‐1 and CXCL10	Immuno‐PET or serum	Positive (+)	Critical for T cell trafficking into the tumor [101, 116]
Distant PD‐L1	Distant biopsy	Negative (−)	Adaptive resistance mechanism. Action: Signal to initiate or intensify PD‐1 blockade [117, 118].
Serum TGF‐β	Serum ELISA	Negative (−)	Drives systemic immunosuppression [61]

*Note*: Candidate biomarkers are organized by immune‐cascade phase.

Abbreviations: cfDNA, cell‐free DNA; CT, computed tomography; ctDNA, circulating tumor DNA; CXCL10, C‐X‐C motif chemokine ligand 10; DCE‐MRI, dynamic contrast‐enhanced magnetic resonance imaging; ELISA, enzyme‐linked immunosorbent assay; ICAM‐1, intercellular adhesion molecule 1; IFN‐I, Type I interferon; IHC, immunohistochemistry; MRI, magnetic resonance imaging; PARP7, poly(ADP‐ribose) polymerase 7; PD‐L1, programmed death‐ligand 1; PET, positron emission tomography; RNA‐seq, RNA sequencing; TCR‐seq, T cell receptor sequencing; TGF‐β, transforming growth factor beta; TMB, tumor mutational burden; Treg, regulatory T cell; WES, whole‐exome sequencing.

## 4 Clinical Translation: Trial Landscape and Evidence

Translating mechanistic insights into clinical reality requires moving beyond case reports to prospective investigation. A challenge that precedes even trial execution is the absence of consensus on what an abscopal response actually is. Current definitions vary widely, resulting in reported incidence rates ranging from under 10% to over 60% [119]. Studies differ in tumor shrinkage thresholds, follow‐up duration, and whether stable disease qualifies as a response. This heterogeneity prevents meaningful cross‐trial comparisons and biomarker validation [120]. Consensus guidelines are needed to define minimum response criteria, standardize follow‐up intervals, and establish reporting requirements for RT parameters and immune biomarkers. Without such standards, systematic study and reliable clinical implementation remain elusive.

As of early 2026, more than 30 trials include the abscopal effect or related systemic immune effects as one of their endpoints. This reflects both active interest and lingering uncertainty [121]. Consistent with the field's exploratory nature, most are Phase II studies spanning strategies from ICI to novel RT techniques [122]. Table 2 summarizes reported clinical trials organized by mechanistic category.

**TABLE 2:** Reported clinical trials of the abscopal effect.

NCT no.	Mechanism of action	Phase	Status	Tumor type	Intervention	Key findings and endpoints
**I. Immune checkpoint inhibitors + RT**						
**A. PD‐1 inhibitors**						
NCT06947694	PD‐1 + multisite RT	II	Recruiting	NSCLC (Stage IV)	Sequential: Chemo‐IO (×4) → multisite RT (5–8 Gy) → IO maintenance	Ongoing trial. Primary endpoint: Optimal RT‐IO sequencing pattern and abscopal effect model development. Novel multisite RT approach.
NCT03480334	PD‐1 (nivolumab) + RT	II	Active, not recruiting	Hodgkin lymphoma (relapsed or refractory, post‐anti‐PD1 progression)	RT to single lesion + nivolumab (4–6 infusions)	Completed enrollment. Primary: Abscopal response rate (ARR‐6) at 12 weeks. Targets patients who previously progressed on anti‐PD1 therapy.
NCT02830594	PD‐1 (pembrolizumab) + RT	II	Completed	Gastroesophageal cancer (Stage IV)	RT + pembrolizumab	Mixed results: Clinical response signals were reported, but biomarker analyses at nonirradiated sites showed no significant changes in CPS (*p* = 0.60), TPS (*p* = 0.64), or MDSC (*p* = 0.87). Study duration: baseline to 105 weeks.
NCT03396471	PD‐1 (pembrolizumab) + RT	II	Terminated	Carcinoma of unknown primary (CUP, Stage IV)	RT + pembrolizumab	Limited efficacy: Abscopal response rate only 7.1% (1 of 14 patients). Indicates this combination has limited efficacy in triggering abscopal effect for CUP patients.
NCT05435053	PD‐1 (nivolumab) + IRE	II	Terminated	Pancreatic cancer (Stage IV)	Irreversible electroporation + nivolumab	Primary: Safety and tolerability (adverse events per CTCAE v4.0) at 6 months. Trial terminated early.
NCT06045286	PD‐1 inhibitors + RT	I	Recruiting	Colorectal cancer (liver metastases, MSS)	High‐dose or low‐dose RT + PD‐1 inhibitors	Primary: Objective response rate (ORR) of low‐dose RT lesions and other metastases by RECIST v1.1 at 1, 3, 6, and 12 months.
NCT06327178	PD‐1 + SBRT	Observational	Recruiting	Renal cell carcinoma (Stage IV)	SBRT + PD‐1 inhibitor	Observational study. Primary: Objective response rate. Follow‐up: Up to 2 years.
**B. PD‐L1 inhibitors**						
NCT04245514	PD‐L1 (durvalumab) + RT	II	Active, not recruiting	NSCLC (Stage III)	RT + durvalumab	Primary: Event‐free survival (EFS) at 12 months (including relapse or progression per RECIST 1.1, second tumor, or death).
NCT03196401	PD‐L1 (durvalumab) + RT	I	Withdrawn	Solitary bone plasmacytoma (prevention of multiple myeloma)	RT + durvalumab	Withdrawn. Planned endpoint: Disease assessment per IMWG criteria including minimal residual disease, up to 36 months.
NCT06953843	PD‐L1 (benmelstobart) + RT ± VEGF	II	Recruiting	NSCLC (Stage IV, EGFR‐ and ALK‐wild‐type)	Variable RT fractions + benmelstobart + chemo ± bevacizumab	Primary: Remission rate of abscopal lesions per RECIST v1.1. Evaluates different RT fractionation modes. Follow‐up: ∼2 years.
**C. CTLA‐4 inhibitors**						
NCT02406183	CTLA‐4 (ipilimumab) + RT	I	Completed	Melanoma (Stage IV)	SBRT + ipilimumab	Completed. Phase I dose‐finding study. Primary: Maximum tolerated dose (MTD) associated with dose‐limiting toxicity (DLT) in 25% of patients. Follow‐up: 2 years.
**D. Dual checkpoint inhibitors**						
NCT03354962	PD‐1 + CTLA‐4 (nivolumab + ipilimumab) + RT	I/II	Terminated	Melanoma (Stage IV)	RT + nivolumab + ipilimumab	Terminated. Phase I: DLT incidence (3 weeks). Phase II: Rate of patients alive without progression at 6 months. Abscopal effect defined as ≥ 20% tumor shrinkage in nonirradiated lesion at 6 weeks.
**II. Cytokines and immune adjuvants + RT**						
**A. GM‐CSF (granulocyte‐macrophage colony‐stimulating factor)**						
NCT03113851	GM‐CSF + RT	II	Completed	NSCLC (Stage IV)	RT (35 Gy in 10 fractions or similar) + rhGM‐CSF	Completed—landmark study. Primary: Abscopal effect rate. Follow‐up: up to 50 months. Part of the seminal Golden et al. work showing ∼27% abscopal response rate with improved OS (21 vs. 8 months in responders vs. nonresponders).
NCT02623595	GM‐CSF + RT	II	Withdrawn	NSCLC (Stage IV, failed second‐line chemotherapy)	SBRT + GM‐CSF	Withdrawn. Planned endpoint: Abscopal effect rate at 4 weeks after rhGM‐CSF completion.
NCT05407649	GM‐CSF + RT	II	Unknown	Thymoma	RT + GM‐CSF	Status unknown. Primary: Abscopal effect rate assessed after treatment initiation, up to 12 months.
NCT02976740	GM‐CSF + T cell activation (thymosin α1) + RT	II	Unknown	NSCLC (Stage IV, failed second‐line chemotherapy)	SBRT + rhGM‐CSF + thymosin alpha‐1	Combination approach. Primary: Abscopal effect rate at 4 weeks after combined treatment completion.
NCT04517539	GM‐CSF + peginterferon alfa‐2b + RT	II	Unknown	Thymic epithelial tumor (Stage IV)	rhGM‐CSF + peginterferon alfa‐2b + RT	Multi‐agent immune adjuvant approach. Primary: Abscopal effect rate, up to 12 months.
**B. Thymalfasin (thymosin α1)**						
NCT02535988	Thymalfasin + RT	II	Withdrawn	Colorectal cancer (Stage IV)	RT + thymalfasin	Withdrawn. Planned endpoint: Proportion of patients with abscopal response at 7–8 weeks.
NCT02542137	Thymalfasin + RT	II	Withdrawn	SCLC (Stage IV)	RT + thymalfasin	Withdrawn. Planned endpoint: Proportion of patients with abscopal response at 7–8 weeks.
NCT02542930	Thymalfasin + RT	II	Withdrawn	NSCLC (Stage IV)	RT + thymalfasin	Withdrawn. Planned endpoint: Proportion of patients with abscopal response at 7–8 weeks.
**III. TGF‐β inhibition + RT**						
NCT01401062	TGF‐β inhibitor (fresolimumab) + RT	II	Completed	Breast cancer (Stage IV)	RT (3 × 7.5 Gy at Week 1, optional second lesion at Week 7) + fresolimumab (1 or 10 mg/kg q3w × 5)	Completed—key findings: Clinical response limited (13% stable disease rate). However, the 10 mg/kg dose showed significantly higher median OS versus 1 mg/kg (HR: 2.73, 95% CI: 1.02–7.30, *p* = 0.039). Higher dose correlated with increased peripheral CD8^+^ T cells and central memory pool. Abscopal response rate was the primary endpoint (assessed at 15 weeks per irRC).
**IV. Thermal ablation + immune adjuvants**						
NCT03993678	Thermal ablation + glycan adjuvant (IP‐001)	I/II	Completed	Advanced solid tumors	Thermal ablation + IP‐001 injection	Completed. Phase I: DLT incidence (Day 1–28). Phase II (melanoma cohort): Disease control (complete response, partial response, or stable disease for at least 12 weeks) per RECIST 1.1 from treatment start.
NCT05688280	Thermal ablation + glycan adjuvant (IP‐001)	I/II	Active, not recruiting	CRC, NSCLC, soft tissue sarcoma (Stage IV)	Thermal ablation + 1.0% IP‐001 for injection	Primary: Safety and tolerability (adverse event incidence) up to 12 weeks. Glycan‐based immune adjuvant approach.
NCT07321847	Microwave ablation + glycan adjuvant (IP‐001)	II/III	Not yet recruiting	Colorectal cancer (liver metastasis)	Microwave tumor ablation + IP‐001 versus ablation alone	Comparative trial. Primary: Progression‐free survival (PFS). PFS defined as time to extrahepatic progression, intrahepatic progression (not amenable to curative locoregional therapy), or death per RECIST v1.1 by BICR. Follow‐up: up to 5 years.
**V. Irreversible electroporation (IRE) + immunotherapy**						
NCT05609656	IRE + CaEP + PD‐1 (pembrolizumab)	II	Terminated	Colorectal cancer (Stage IV)	Calcium electroporation (CaEP) + IRE + pembrolizumab	Terminated. Primary: Adverse event incidence rate per CTCAE v4.0, up to 1 month after treatment end. Novel electroporation‐based approach.
**VI. Radiotherapy techniques (RT alone)**						
NCT04168320	SBRT‐PATHY	I	Terminated	Bulky tumors	SBRT‐PATHY (SBRT‐based partial tumor irradiation targeting hypoxic segment)	Terminated. Primary: Bystander and abscopal effects. Rates of ≥ 30% tumor regression in partially treated bulky tumors (bystander) and unirradiated oligometastases or regional nodes (abscopal). Novel hypoxia‐targeted partial tumor RT technique.
NCT05578274	LDRT + SBRT	N/A	Not yet recruiting	Neoplasms with secondary malignant neoplasm	SBRT + low‐dose RT (LDRT)	Primary: Abscopal effect rate of low‐dose RT lesions at 3 months after RT completion. Exploring low‐dose RT phenomenon.
NCT05733156	SBRT + LDRT	N/A	Recruiting	Neoplasms with secondary malignant neoplasm	High‐dose RT (HDRT) + low‐dose RT (LDRT)	Primary: Abscopal effect rate of LDRT lesions assessed by RECIST v1.1 at 3 months after RT completion. Dual‐dose RT approach.
NCT07240610	RT + ICIs (general)	Observational	Recruiting	Renal cell carcinoma (Stage IV)	RT + ICIs (various)	Observational study. Primary: Abscopal response rate (ARR) at 1 year after image‐guided ultra‐hypofractionated RT (IGU). ARR defined as ≥ 30% reduction in sum of diameters of nonirradiated lesions (RECIST 1.1) confirmed by central review.
**VII. Other combination therapies**						
**A. Checkpoint inhibitors + VEGF inhibitors + RT**						
NCT04238169	PD‐1 + RT ± VEGF (toripalimab ± bevacizumab)	II	Completed	NSCLC (Stage IV, non‐squamous)	SBRT + toripalimab ± bevacizumab	Completed. Primary: Objective response rate (ORR) per RECIST 1.1 and EORTC 1999 at 6 months. Secondary: Objective response of nontarget lesions. Evaluates VEGF inhibition in addition.
**B. Radiopotentiators + chemo‐IO**						
NCT04873440	RT + MnCl_2_ + chemo‐IO	I/II	Unknown	Lymphoma or solid tumors (Stage IV)	RT + manganese chloride + chemo‐immunotherapy	Phase I/II trial. Primary: Proportion with abscopal response (≥ 30% decrease in longest diameter of nonirradiated lesion) at 6 months. Secondary: Treatment‐related AEs per CTCAE v5.0 at 12 months. Manganese as radiosensitizer.
**C. RT + tyrosine kinase inhibitors**						
NCT02334709	RT + TKIs	I	Completed	Renal cell carcinoma (Stage IV)	SBRT + pazopanib	Completed. Primary: Dose‐limiting toxicity before TKI start, before SBRT, at SBRT end, and each follow‐up. Follow‐up: 2 years. Evaluates RT‐TKI combination safety.
**D. Chemoradiotherapy approaches**						
NCT02474186	Chemo‐RT	II	Completed	Breast cancer or solid tumors (Stage IV)	RT + GM‐CSF	Completed. Primary: Proportion with abscopal response at 7–8 weeks. Chemo‐RT combination without immunotherapy.
NCT03323424	RT + immunomodulator (ADCC‐based)	II	Withdrawn	Colorectal, breast, and head and neck cancers (Stage IV)	SBRT + systemic treatment (ADCC‐capable therapies)	Withdrawn. Planned endpoints: PFS per RECIST v1.1 at disease‐specific timepoints (breast: 18 months, head and neck: 6 months, CRC: 9 months). Novel ADCC‐based immunomodulation approach.

*Note*: Data source: ClinicalTrials.gov (https://clinicaltrials.gov/; accessed February 11, 2026).

Abbreviations: ADCC, antibody‐dependent cellular cytotoxicity; ARR, abscopal response rate; BICR, blinded independent central review; CaEP, calcium electroporation; CPS, combined positive score; CRC, colorectal cancer; CTCAE, Common Terminology Criteria for Adverse Events; CTLA‐4, cytotoxic T‐lymphocyte‐associated protein 4; DLT, dose‐limiting toxicity; EFS, event‐free survival; EORTC, European Organisation for Research and Treatment of Cancer; GM‐CSF, granulocyte‐macrophage colony‐stimulating factor; HDRT, high‐dose radiotherapy; ICI, immune checkpoint inhibitor; IO, immunotherapy; IRE, irreversible electroporation; irRC, immune‐related response criteria; LDRT, low‐dose radiotherapy; MDSC, myeloid‐derived suppressor cell; MSS, microsatellite stable; NSCLC, non‐small cell lung cancer; ORR, objective response rate; PD‐1, programmed death‐1; PD‐L1, programmed death‐ligand 1; PFS, progression‐free survival; RECIST, Response Evaluation Criteria in Solid Tumors; RT, radiotherapy; SBRT, stereotactic body radiotherapy; SCLC, small‐cell lung cancer; TGF‐β, transforming growth factor beta; TKI, tyrosine kinase inhibitor; TPS, tumor proportion score.

A striking feature of this landscape is the high withdrawal rate. Roughly one in three registered trials has been terminated early. This highlights the persistent difficulty of inducing measurable responses in heterogeneous populations [123]. This pattern warrants attention. It speaks less to biological implausibility than to practical challenges of trial execution. Major hurdles include stringent eligibility criteria, the absence of biomarkers, and binary endpoint definitions that may obscure systemic benefits [124].

### 4.1 ICI: Promise Tempered by Heterogeneity

T cell reinvigoration is central to the abscopal mechanism. PD‐1 and PD‐L1 combinations dominate the field, accounting for roughly a third of all registered studies. The results have been instructive yet mixed. Chao et al. (2022) reported a 29% response rate in metastatic gastroesophageal cancer using pembrolizumab with RT [125]. While some patients achieved durable responses, investigators could not distinguish definitive abscopal changes from systemic activity. This highlights a recurring challenge in single‐arm designs lacking immunotherapy‐only controls [125]. Similarly, a Phase II trial in carcinoma of unknown primary terminated early after finding a response in only one of 14 patients. These data do not imply the pairing is ineffective. Rather, they underscore that success is not universal. Baseline tumor immune contexture is likely the critical determinant. Preclinical data reinforce this view, showing that immune deserts resist abscopal induction even when RT is optimally delivered [123].

Several ongoing trials address these limitations through more targeted strategies. NCT06947694 tests a sequential approach in NSCLC where chemo‐immunotherapy induction precedes RT. This design aims to prime systemic immunity before applying the radiation stimulus. NCT06953843 takes a mechanistic angle. It compares fractionation regimens with benmelstobart to determine if fraction size modulates abscopal potential. NCT04245514 extends this inquiry in Stage III lung cancer by explicitly measuring distant disease control. CTLA‐4 blockade has also been explored. NCT02406183 established the safety of ipilimumab with SBRT in melanoma, providing key dose‐escalation data. However, a subsequent triple‐modality study halted early. The addition of nivolumab raised significant concerns regarding the toxicity of intensive immune costimulation.

### 4.2 Cytokine Strategies and TGF‐β Blockade

An important clinical proof‐of‐concept for a deliberately engineered abscopal response comes from the granulocyte‐macrophage colony‐stimulating factor (GM‐CSF) era. Golden et al. (2015) reported that adding GM‐CSF to palliative RT yielded an abscopal response in approximately 27% of evaluable patients [126]. Responders achieved a median OS of 21 months compared with 8 months in nonresponders. This association positioned abscopal response as a potential surrogate for survival benefit [126]. Consequently, this study remains an important clinical study. It suggested that immunological amplification may be needed to convert rare spontaneous responses into a more reproducible phenomenon. Efforts to extend this success using thymalfasin‐based strategies have been less rewarding. Multiple trials were withdrawn before completion. This reflects the practical difficulty of sustaining enrollment in trials that require patients to have accessible and measurable extra‐focal lesions.

Another contribution comes from TGF‐β inhibition. Formenti et al. (2018) investigated fresolimumab combined with RT in metastatic breast cancer [127]. Although radiographic abscopal responses were modest, patients treated at the higher dose showed significantly longer OS. This benefit was accompanied by measurable increases in peripheral CD8^+^ T cells and central memory expansion. The dissociation between radiographic regression and immunological activation suggests that some immune effects may not be captured by binary tumor‐shrinkage endpoints. This supports incorporating immune biomarkers and micrometastasis‐sensitive imaging into future trials while avoiding overinterpretation of radiographic nonresponse.

### 4.3 Novel Technical Approaches and the Path Forward

Two technical strategies are under clinical exploration. First, low‐dose RT is being tested as an immunological adjuvant. Using sub‐ablative doses, this approach aims to reprogram the TME through macrophage polarization and vascular normalization. Preclinical evidence suggests that it may sensitize distant sites to effector T cells mobilized by synchronous high‐dose treatment [128]. NCT05733156 translates this rationale into the clinic by combining high‐dose and low‐dose RT to test whether immune priming increases abscopal potential. Second, thermal ablation combined with glycan‐based immune adjuvants uses ablation‐derived tumor debris as an in situ antigen source. NCT03993678 established the safety of this approach, and NCT07321847 will compare microwave ablation with and without IP‐001. These approaches remain investigational until randomized efficacy data are available.

Clinical evidence supports several practical conclusions. Abscopal responses can occur when RT is paired with immunological amplification, but they remain limited to a minority of patients and vary across tumor types. High trial withdrawal rates also caution against broad enrollment of unselected populations [123]. Future studies should pair response assessment with biomarker development so that likely responders can be identified prospectively, while keeping RT reporting and immune correlative measurements aligned across trials [124].

Together, these clinical data show that translation is possible but inconsistent, making the optimization of radioimmunotherapy combinations an important next step.

## 5 Optimizing Radioimmunotherapy Synergy

Kodama et al. (2013) reported two cases from 1999 and 2009 in which patients receiving RT combined with *Bacillus* Calmette–Guérin (BCG) cell wall skeleton exhibited abscopal effects [129]. The 1999 case is regarded as an early documented instance where RT combined with immunotherapy triggered such an effect. With the widespread adoption of ICIs, however, the frequency of reported abscopal effects has risen substantially. In our systematic review of 177 cases, 86 involved immunotherapies, most of which were reported after 2017 (Table S1). This trend is consistent with clinical observations [130] and preclinical research, indicating that RT can initiate immune responses, whereas ICIs can relieve inhibitory checkpoints and thereby facilitate systemic tumor control in selected contexts. Table 3 summarizes how major immunotherapeutic partners can support different stages of the RT‐induced immune cascade.

**TABLE 3:** Synergy between radiotherapy and immunotherapy for abscopal effect.

Class	Targeted phase	Mechanism	Evidence	Safety
ICIs (PD‐1 and PD‐L1, CTLA‐4)	Orchestration and execution [131, 132]	− Enhance priming and epitope spreading − Restore effector T cell function	Abscopal regressions in melanoma, NSCLC; PEMBRO‐RT improved out‐of‐field response rates	Pneumonitis (thoracic RT), radionecrosis (CNS RT), lymphopenia
Cytokines (GM‐CSF, IL‐2, IL‐12, etc.)	Orchestration and execution [12]	− Expand effector pools − Boost Th1 signals, CTL and NK cytotoxicity	RT + GM‐CSF: ∼26%–27% abscopal responses; early IL‐2 + SBRT reports	High‐dose IL‐2 systemic toxicity (vascular leak); lymphopenia
Costimulatory agonists (OX40, 4‐1BB, CD27, CD40)	Orchestration [133]	− Amplify T cell expansion − Enhance DC licensing	Preclinical synergy strong; limited human evidence	Immune‐related AEs, sparse RT‐specific toxicity data
Oncolytic viruses (OVs)	Ignition [134, 135]	− RT enhances viral entry and spread − OVs act as in situ vaccines	Case reports + reviews; mostly in ICI combinations	Expected OV toxicities; RT‐site inflammation may worsen local toxicity
Therapeutic vaccines (neoantigen and DC)	Ignition and orchestration [136, 137]	− RT supplies antigen and danger signals − Promotes epitope spreading	RT + PSA vaccine: Elevated PSA‐specific T cell frequency; DC intratumoral + RT showed immune responses	Generally manageable RT‐ and vaccine‐related AEs; few high‐grade events
Adoptive cell therapies (TILs, TCR, CAR)	Execution [138]	− Increased antigen density and MHC‐I expression − Facilitate T cell homing	RT + adoptive T cells durable responses in mice; TIL + TBI increased toxicity without benefit	Tissue inflammation; TBI toxicities (e.g., thrombotic microangiopathy)

*Note*: Immunotherapeutic partners are summarized according to their putative role in the RT‐induced immune cascade.

Abbreviations: AE, adverse event; CAR, chimeric antigen receptor; CNS, central nervous system; CTL, cytotoxic T lymphocyte; CTLA‐4, cytotoxic T‐lymphocyte‐associated protein 4; DC, dendritic cell; GM‐CSF, granulocyte‐macrophage colony‐stimulating factor; ICI, immune checkpoint inhibitor; IL, interleukin; MHC‐I, major histocompatibility complex class I; NK, natural killer; OV, oncolytic virus; PD‐1, programmed death‐1; PD‐L1, programmed death‐ligand 1; PSA, prostate‐specific antigen; RT, radiotherapy; TBI, total body irradiation; TCR, T‐cell receptor; TIL, tumor‐infiltrating lymphocyte.

In this section, the focus shifts from mechanism to practical optimization, including drug selection, timing, and safety.

### 5.1 Selection of Immunotherapy: From Single Target to Multi‐Blockade

Currently, immunotherapies combined with RT are predominantly ICIs, with PD‐1 and PD‐L1 antibodies being the most widely used. Multiple studies report that PD‐1 and PD‐L1 inhibitors combined with RT achieve systemic objective response rates of approximately 23%–38% across solid tumors [40, 139, 140, 141, 142, 143]. In selected studies, this approach has shown higher out‐of‐field response rates than immunotherapy alone [144]. The Phase II PEMBRO‐RT trial provided randomized evidence. In metastatic NSCLC patients, the 12‐week objective response rate was 36% with pembrolizumab plus SBRT, double that of the control group. Moreover, both PFS and OS improved, with benefits particularly pronounced in the PD‐L1 negative subgroup [140]. Collectively, these data support anti‐PD‐1 and PD‐L1 agents as a commonly used strategy in radioimmunotherapy, given their favorable balance of efficacy and safety [145].

Mechanistic studies support this pairing because RT can induce PD‐L1 expression and PD‐1 or PD‐L1 blockade can counter adaptive immune resistance [146, 147, 148].

Targeting the CTLA‐4 pathway offers another route for synergy, acting primarily during the early phase of T cell activation. Preclinical studies confirm the synergistic potential of anti‐CTLA‐4 antibodies (e.g., ipilimumab) when combined with RT [85]. Clinical case reports indicate that this combination has induced abscopal effects in multiple tumor types, including melanoma [80, 149], RCC [150], NSCLC [151], head and neck cancer [152, 153], and chordoma [154]. Individual case reports have described complete remission after combined checkpoint blockade and RT in patients with prior therapeutic resistance [155].

To increase immune activation, strategies combining dual checkpoint inhibition (anti‐PD‐1 plus anti‐CTLA‐4) with RT are actively under investigation. For example, the ongoing SAMURAI trial [156] is evaluating nivolumab plus ipilimumab combined with SBRT in RCC. However, efficacy improvements are often accompanied by increased toxicity. A meta‐analysis reported that dual ICI therapy combined with SBRT improved 6‐month OS but also significantly increased the risk of treatment‐related adverse events [157]. This intensified strategy may therefore be more suitable for patients with selected highly immunogenic or refractory tumors. Clinical decisions must carefully balance anticipated benefits against potential toxicities.

Combination escalation is being explored for tumors that do not respond to RT plus a single ICI. Adding chemotherapy, targeted therapy, or an immunomodulator may help convert immunologically “cold” tumors into more permissive immune environments. In preclinical models, a single low dose of liposomal doxorubicin added to RT plus anti‐PD‐1 enhanced the abscopal effect through the mitochondrial DNA (mtDNA) activation of the cGAS–STING pathway and increased cross‐presenting DCs and tumor‐specific CD8^+^ T cells in distant lesions [158]. A 2025 NSCLC case report is consistent with this concept, but remains anecdotal evidence rather than clinical validation [146]. Agents such as colony‐stimulating factor 1 receptor (CSF1R) inhibitors, CD40 agonists [159], and cGAS–STING agonists [160, 161] should therefore be presented as candidates for prospective testing, not as established clinical approaches.

Other investigational partners remain less mature and should be tested prospectively: Checkpoint modulation can reshape tumor‐specific T cell clones [162]; costimulatory or cytokine‐like strategies may support effector expansion [163, 164]; oncolytic viruses can activate innate pathways [165, 166]; and RT‐derived microparticles may enhance MHC‐I expression [167].

### 5.2 Treatment Timing: Balancing Sequential and Concurrent Approaches

Beyond drug selection, the timing of RT and immunotherapy administration represents another important determinant of synergistic efficacy, though no universal optimal strategy has yet been established.

In unresectable Stage III NSCLC, sequential therapy has become the standard of care. The PACIFIC trial demonstrated that durvalumab consolidation administered from 1 to 42 days after concurrent chemoradiotherapy significantly improved PFS and OS [168, 169, 170]. Additional studies suggest that initiating immunotherapy earlier after RT (e.g., within 1 week) may be more effective than delayed administration [171]. A recent Phase II trial in head and neck cancer with 4‐year follow‐up similarly indicated that sequential pembrolizumab after chemoradiation yielded superior survival outcomes compared to concurrent administration [172].

Conversely, the rationale for concurrent administration lies in introducing ICIs during the peak window of RT‐induced antigen release and inflammatory responses, thereby maximizing immune amplification. A retrospective NSCLC study published in 2024 reported that concurrent ICI plus chemoradiotherapy achieved a higher objective response rate (29.4% vs. 23.4%) and disease control rate (49.0% vs. 38.3%) compared with the sequential approach [173]. In metastatic disease lacking effective local control options, this approach is particularly attractive, as it aims to induce systemic immune responses from the outset of therapy.

The optimal timing strategy is likely to depend on tumor type, chemotherapy regimen, ICI subtype, and host immune reserve. Prospective trials should define context‐specific schedules rather than assuming that concurrent or sequential treatment is uniformly superior.

### 5.3 Safety and Practical Considerations

Systemic immune activation underlying the abscopal effect often manifests clinically as generalized inflammation or autoimmunity. For example, patients receiving combined radioimmunotherapy can develop immune‐related adverse events (irAEs) such as bullous pemphigoid, which may coincide with the abscopal effect [174]. This is further illustrated by the “abscopal radiation recall” phenomenon. In one case, reirradiation in a patient receiving nivolumab triggered a widespread rash far beyond the treatment field, indicating a systemically hypersensitized immune state [60]. Preexisting autoimmune conditions may lower the threshold for inducing an abscopal effect. For instance, a patient with autoimmune hepatitis responded to a single fraction of palliative RT [175].

More intensive combination strategies are often accompanied by higher toxicity risks. As summarized in Table S2, the complexity of combination regimens is directly associated with an increased risk of severe toxicities. Data from a meta‐analysis indicated that compared with SBRT plus monotherapy ICI, dual ICI therapy (anti‐PD‐1 or anti‐PD‐L1 plus anti‐CTLA‐4) nearly tripled the risk of Grade ≥ 3 adverse events (32% vs. 11%). In patients with unresectable hepatocellular carcinoma (HCC), triple therapy incorporating targeted agents demonstrated the highest toxicity burden, with the overall incidence of severe adverse events exceeding 60%. Although largely driven by manageable hematologic toxicities, the risk remained substantially greater than that of no RT control groups.

However, these intensive regimens raise critical feasibility and ethical concerns for frail, late‐stage patients. Frail patients (PS ≥ 2) often derive minimal survival benefit from aggressive combinations while experiencing disproportionate toxicity [176]. Geriatric assessment tools should guide patient selection. Functional reserve, rather than chronological age, determines tolerability [177]. Ethically, treatment decisions must balance lifespan extension against quality‐of‐life preservation. For patients with a prognosis limited to 6 or 12 months, de‐escalated approaches using RT alone or paired with monotherapy are often appropriate. These strategies may better align with patient‐centered goals [178]. These data support rigorous toxicity monitoring and management [157, 179, 180].

In summary, optimization of radioimmunotherapy synergy requires matching the immunotherapeutic partner, treatment sequence, and toxicity profile to the biological and clinical context. This need for careful balance is particularly important because systemic immune effects after RT are not uniformly beneficial.

## 6 Beyond Positive Effects: The Badscopal Phenomenon

**FIGURE 4:**
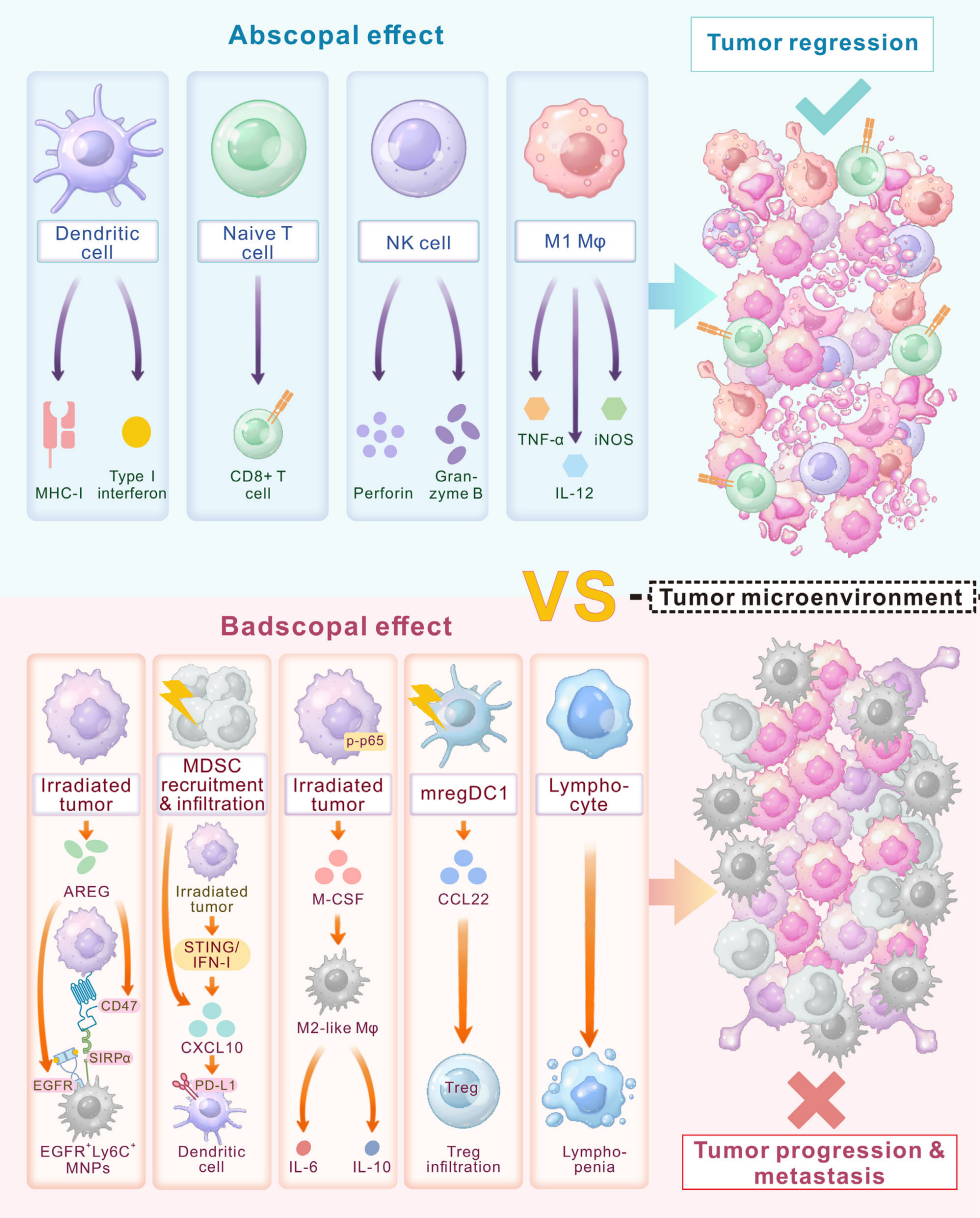
Radiotherapy drives opposing systemic immune outcomes: abscopal versus badscopal. RT activates competing immune pathways within the TME that determine distant tumor fate. Effector pathways (blue) drive the abscopal effect via DC activation, CD8^+^ T cell priming, NK cell cytotoxicity, and M1 TAM polarization, collectively promoting tumor regression. Suppressive pathways (red) drive the badscopal effect through five axes: AREG–EGFR–CD47 signaling, PD‐L1 upregulation with MDSC accumulation, NF‐κB (p65)‐M‐CSF myeloid immunosuppression, CCL22–FOXP3^+^ Treg‐mediated cDC1 inhibition, and RT‐induced lymphopenia, collectively promoting distant metastasis. The balance of these forces within the TME battlefield determines clinical outcome.

Current evidence for the badscopal phenomenon remains strongest in preclinical and translational models, with limited clinical validation. Therefore, the mechanisms discussed below should be interpreted as candidate suppressive trajectories rather than established clinical pathways. Although RT can trigger systemic antitumor immunity, some preclinical and translational studies suggest that local irradiation may also contribute to out‐of‐field tumor progression under selected conditions [11]. This adverse systemic trajectory has been termed the “badscopal” effect. Candidate mechanisms can be grouped into several interacting processes. These include amphiregulin (AREG)–epidermal growth factor receptor (EGFR)–cluster of differentiation 47 (CD47) myeloid reprogramming with impaired phagocytosis and PD‐L1 upregulation with MDSC accumulation [181, 182, 183, 184]. These opposing systemic trajectories are illustrated in Figure 4 and compared in Table 4.

**TABLE 4:** Abscopal versus badscopal responses to radiotherapy.

Feature	Abscopal effect	Badscopal effect
**Primary outcome**	Systemic regression of nonirradiated lesions [4, 120, 121]	Possible acceleration of distant metastases [11, 185, 186]
**Key immune effectors**	CD8^+^ cytotoxic T cells, NK cells, M1 macrophages [19, 42, 99]	MDSCs, M2 macrophages, Tregs, regulatory DCs [11, 182, 183, 184, 187, 188]
**Critical cytokines and signals**	Type I IFN, local CXCL10 (T cell recruiting), IL‐12 [19, 99, 101]	AREG–EGFR–CD47, systemic CXCL10–PD‐L1–MDSC, M‐CSF cascade, CCL22–Treg–CD86, supplemental YTHDF2–Notch–MHC‐I DC‐intrinsic axis [11, 182, 183, 184, 187, 188, 189]
**Myeloid programming**	Enhanced antigen presentation via cDC1 activation [19, 30, 42]	AREG‐driven M2 polarization, ATR–CD47 axis blocks phagocytosis, PD‐L1 upregulation on DCs [11, 182, 189, 190]
**Adaptive response**	Epitope spreading, T cell priming [31, 40, 99, 103]	T cell exhaustion, CCL22‐mediated Treg recruitment, CD86‐driven Treg expansion, lymphopenia [93, 94, 184, 188]
**Impact of lymphocyte count**	Requires preserved absolute lymphocyte count [93, 94]	Associated with severe, prolonged RT‐induced lymphopenia [93, 94, 103]
**Candidate experimental strategies**	Checkpoint blockade, RT dose and field optimization, and investigational STING‐pathway activation [85, 87, 140, 160, 161]	Lymphocyte‐sparing RT and prospective testing of pathway‐directed combinations; no single intervention is established [93, 94, 182, 184, 187, 188, 189, 191]

*Note*: Abscopal and badscopal features are compared using representative peer‐reviewed evidence.

Abbreviations: AREG, amphiregulin; CCL22, C‐C motif chemokine ligand 22; CD47, cluster of differentiation 47; CD86, cluster of differentiation 86; cDC1, conventional Type 1 dendritic cell; DC, dendritic cell; EGFR, epidermal growth factor receptor; IFN, interferon; IL, interleukin; M‐CSF, macrophage colony‐stimulating factor; MDSC, myeloid‐derived suppressor cell; MHC‐I, major histocompatibility complex class I; NK, natural killer; PD‐L1, programmed death‐ligand 1; RT, radiotherapy; STING, stimulator of interferon genes; Treg, regulatory T cell; YTHDF2, YTH N6‐methyladenosine RNA‐binding protein 2.

The AREG–EGFR–CD47 axis is currently one of the best‐characterized proposed mechanisms. Piffkó et al. reported that localized high‐dose RT increased AREG expression in tumor cells, which then promoted EGFR‐dependent myeloid reprogramming and CD47 upregulation on tumor cells. This pathway suppressed macrophage phagocytosis and was associated with expansion of nonirradiated metastases in experimental models [11]. In clinical cohorts receiving SBRT, elevated circulating AREG correlated with metastatic progression and inferior survival [11]. Mechanistically, AREG may act on EGFR‐expressing myeloid cells to drive M2 polarization and may upregulate CD47 through ATR‐mediated DNA damage responses [189]. EGFR signaling can further stabilize CD47 through Src [192] or promote its transcription through STAT3 [193], suggesting a potential systemic barrier to antitumor immunity.

Complementary myeloid and adaptive circuits may amplify this suppressive trajectory. Although CXCL10 is usually considered important for recruiting effector T cells to tumors, recent findings suggest a context‐dependent adverse role after RT. RT can drive systemic MDSC mobilization, and RT‐induced CXCL10 signaling can promote PD‐L1 upregulation on DCs, thereby supporting distant metastases in experimental models [182, 194]. Blocking PD‐L1, CXCL10, or MDSCs reduced this effect [182, 194]. RT can also trigger M‐CSF release from tumor cells through p65 phosphorylation, enhancing M‐CSF transcription and amplifying suppressive macrophage programs [183].

An additional DC‐intrinsic mechanism outside the five candidate badscopal axes involves radiation‐associated suppression of DC antitumor function through epigenetic mechanisms. Chen et al. (2026) showed that ionizing radiation induced YTH N6‐methyladenosine RNA‐binding protein 2 (YTHDF2), an m6A reader protein, in DCs through SPI1‐mediated transcription. Elevated YTHDF2 promoted m6A‐dependent degradation of Notch pathway regulators, impaired MHC‐I cross‐presentation, and reduced CD8^+^ T cell activation. In the COSINR trial (NCT03223155), YTHDF2 upregulation in DCs correlated with disease progression. Genetic or pharmacological YTHDF2 inhibition restored antigen cross‐presentation and reduced metastases across multiple tumor models, identifying YTHDF2 as a candidate radiation‐induced immune checkpoint in DCs [187].

Beyond myeloid reprogramming, RT can expand conventional Type 1 DC (cDC1) populations that secrete C‐C motif chemokine ligand 22 (CCL22) and recruit Tregs [184]. In lymphocyte‐depleted contexts, PD‐1 or CTLA‐4 blockade may paradoxically enhance Treg responses through CD28 costimulation, whereas cluster of differentiation 86 (CD86) blockade limits Treg expansion and improves tumor control [188]. RT‐induced lymphopenia provides a plausible link between these circuits. Modern clinical data suggest that RT‐induced lymphopenia can give numerical and functional advantages to Tregs and suppressive myeloid cells, thereby increasing the risk of unfavorable systemic immune remodeling [195, 196, 197].

These proposed mechanisms contrast with classical abscopal responses but share upstream triggers, including DNA damage, cGAS–STING activation, and DAMP and antigen release. Abscopal trajectories favor effective DC cross‐priming and CD8^+^ T‐cell and NK‐cell responses through balanced IFN signaling [198]. Badscopal trajectories instead involve PD‐L1 and CD47 amplification through ATR or cGAS–STING, expansion of suppressive myeloid populations and Tregs, and lymphopenia with skewed myeloid‐to‐lymphoid ratios [195, 199, 200]. RT parameters may influence this divergence: Moderate doses with limited volumes preserve lymphocytes and can support abscopal responses, whereas high doses, large fields, or prolonged fractionation may promote lymphopenia and pro‐metastatic mediator release [103, 201]. A comparison of abscopal and badscopal features is provided in Table 4.

Clinical evidence remains limited and should be interpreted cautiously. Koukourakis et al. (2020) reported a bladder cancer patient who experienced complete regression of irradiated lesions under RT plus anti‐PD‐1 therapy, followed by rapid development of sternum and liver metastases outside the radiation field [185]. More recently, Chiang et al. (2026) identified CD14 expression in urothelial cancer cells as a potential predictor of RT‐promoted distant metastasis. CD14‐driven NF‐κB activation upregulated neutrophil‐recruiting chemokines such as CXCL1, CXCL2, and CXCL3, creating a neutrophil‐enriched TME associated with metastatic susceptibility after RT. Targeting CD14 or blocking neutrophil infiltration through CXCR2 antagonism reduced RT‐promoted metastasis in preclinical models [186]. Because lymphopenia, tumor heterogeneity, and treatment selection may confound these observations, current data support the badscopal effect as a hypothesis requiring prospective clinical validation rather than a settled causal phenomenon.

Local RT can also affect the systemic immune compartment. Lymphocytes are among the most radiosensitive hematopoietic cells, with an LD50 of 1–2 Gy [103, 201], and incidental irradiation of lymphocyte‐rich organs such as the spleen, thymus, bone marrow, and TDLNs can cause measurable systemic depletion. Radiation‐induced lymphopenia develops in 40%–70% of patients, with lymphocyte counts recovering to a median of only 55% of baseline within 1 year, and is associated with inferior survival across tumor types [103, 201]. These findings support treating lymphoid organs and circulating immune cells as organs at risk during RT planning. Spleen‐sparing techniques and beam‐on time reduction may represent practical near‐term mitigation strategies, complementing the lymphocyte‐sparing rationale for proton therapy discussed in Section 3.2.

Clinical translation remains challenging: CD47 blockade has encountered Phase III setbacks related to anemia and limited efficacy [202, 203], whereas EGFR inhibitors combined with thoracic RT may increase the risk of severe interstitial lung disease [204, 205]. These limitations argue against presenting CD47 blockade, EGFR inhibition, or any single intervention as an established badscopal countermeasure. Because badscopal mechanisms involve multiple suppressive axes, CD47 inhibition alone may leave parallel circuits intact. Multi‐targeted combinations or next‐generation bispecific antibodies should therefore be framed as future directions for prospective testing rather than validated clinical approaches [191]. Multicenter clinical and translational studies are needed to determine whether the badscopal effect is reproducible in patients and how it should influence RT‐immunotherapy strategies.

## 7 Conclusion, Unanswered Questions, and Future Perspectives

Cumulative evidence indicates that the abscopal effect is not a universal property of RT but a rare, context‐dependent outcome shaped by tumor immunogenicity, microenvironmental resistance, RT design, and host immune reserve. RT acts primarily as an immune primer. Consistent systemic control usually requires suitable radiation parameters, preserved lymphocyte function, and an immunotherapeutic partner matched to the biological context. The badscopal effect further cautions that RT‐induced immune modulation is not uniformly beneficial and should be studied with the same rigor as favorable systemic responses.

A critical insight emerging from this synthesis is that the abscopal and badscopal effects share identical upstream triggers, including DNA damage, cGAS–STING activation, and DAMP release, yet diverge sharply at the immunological checkpoint between effective cross‐priming and suppressive myeloid reprogramming. This divergence is shaped by factors such as RT dose and field size, baseline lymphocyte reserve, and the myeloid‐to‐lymphoid balance within the TME. The field has historically underappreciated this bidirectionality, leading to trial designs that select patients broadly, measure only radiographic abscopal responses, and fail to monitor suppressive trajectories in parallel.

A randomized trial in head and neck cancer illustrates this limitation. In metastatic HNSCC, adding SBRT to nivolumab did not improve response and did not provide evidence of an abscopal effect in an unselected population, underscoring that systemic RT–ICI synergy cannot be assumed without biological stratification [206]. The main challenge is no longer demonstrating that the abscopal effect can occur, but rather identifying the precise conditions under which it reliably does so.

Future work should focus on the points that determine divergence between response and resistance: The timing and intensity of cGAS–STING and IFN‐I signaling, the division of labor between T cells and macrophages during execution, and the suppressive circuits that may drive badscopal trajectories.

Biomarker development should therefore prioritize dynamic readouts rather than isolated baseline markers [122]. Serial TCR profiling, functional imaging, and immune‐cell state analysis may help determine whether RT is promoting systemic priming or suppressive remodeling after treatment initiation.

Clinically, the field must move to prospective, biomarker‐integrated trial designs. These should stratify patients by baseline immunogenicity and incorporate adaptive endpoints capable of capturing both immune activation and suppressive signals. Optimal radiation dose, timing, and fractionation for inducing the abscopal effect remain undefined. Combining RT with immunotherapy introduces complex challenges in sequencing and toxicity management that require prospective resolution [207].

Technologically, next‐generation modalities warrant rigorous comparative evaluation. These include FLASH RT [208, 209], spatially fractionated approaches [210], and metabolic‐targeting combinations [211]. Each may modulate the immunosuppressive TME through distinct mechanisms. Comparative designs should also capture systemic immune activation and suppressive trajectories alongside radiographic outcomes.

Ultimately, making the abscopal effect a predictable therapeutic tool will require integrating mechanistic discovery, biomarker validation, and rational trial design. This effort must address both beneficial and detrimental systemic immune trajectories with equal rigor.

## Author Contributions

Y.D. conducted the literature search, prepared the figures, and drafted the manuscript. M.C., X.H., and X.T. assisted with the literature search and data organization. X.Y. contributed to revisions and supervision. J.L. conceived the topic, provided critical revisions, integrated the content, and supervised the overall preparation of the review. All authors approved the final manuscript.

This work was supported by the National Natural Science Foundation of China (Grants 82372945, 82072716, 82573145, 82473255, 82102829, 82003231), the Key Clinical Specialty Project of Shanghai, and the National Key Research and Development Program of China (No. 2023YFC2413900).

## Ethics Statement

The authors have nothing to report.

## Conflicts of Interest

The authors declare no conflicts of interest.

## Supporting information




**Supporting File 1**: mco270932‐sup‐0001‐TableS1.xlsx

**Supporting File 2**: mco270932‐sup‐0002‐TableS2.docx

## Data Availability

The authors have nothing to report.
