# Supplementary material for: Abscopal Effect in Radiation Therapy: The Immune Mechanisms and Clinical Advances
Source: MedComm (2020). 2026 Aug 17;7(9):e70932. doi: 10.1002/mco2.70932 (PMC13482354; doi:10.1002/mco2.70932)
Supplement: Supplementary file 1 [file MCO2-7-e70932-s002.docx]

# Abscopal Effect in Radiation Therapy: The Immune Mechanisms and Clinical Advances

Yihong Dong^1,2^, Moran Chen^1,3^, Xupeng Hou^1,3^, Xiaoya Tang^1^, Xiaoli Yu^4*^, Jing Liu^1*^

1. Key Laboratory of Breast Cancer in Shanghai, Department of Breast Surgery, Fudan University Shanghai Cancer Center, Shanghai 200032, China; Department of Oncology, Shanghai Medical College, Fudan University, Shanghai 200032, China.

2. Department of Clinical Medicine, Wuxi School of Medicine, Jiangnan University, Wuxi 214000, China.

3. Department of Pancreatic Cancer, Tianjin Medical University Cancer Institute and Hospital, National Clinical Research Center for Cancer; Key Laboratory of Cancer Prevention and Therapy, Tianjin’s Clinical Research Center for Cancer, Tianjin 300060, China.

4. Department of Radiation Oncology, Fudan University Shanghai Cancer Center, Shanghai, China; Department of Oncology, Shanghai Medical College, Fudan University, Shanghai, China; Shanghai Clinical Research Center for Radiation Oncology, Shanghai Key Laboratory of Radiation Oncology, Shanghai 200032, China.

Grant support: This work was supported by the National Natural Science Foundation of China (Grants 82372945, 82072716, 82573145), the Collaboration Program of Beijing Tianjin and Hebei (Grant 22JCZXJC00120), and the Key Clinical Specialty Project of Shanghai.

Correspondence to Jing Liu (piano_solo@163.com) and Xiaoli Yu (xiaoliyu@fudan.edu.cn)

##### **Supplementary Table S2.** Grade ≥3 Treatment-Related AEs with Combined RT and Systemic Therapy

| **Treatment Regimen** | **Patient Population** | **Incidence of Grade ≥3 AEs, % (n of N)** | **Ref.** |
| --- | --- | --- | --- |
| SBRT + Anti-PD-1 or Anti-PD-L1 Monotherapy | Metastatic solid tumors (pooled from prospective trials) | 11% (95% CI 5% to 17%) | Urias et al.^1^ |
| SBRT + Dual ICI (anti-PD-1 or anti-PD-L1 plus anti-CTLA-4) | Metastatic solid tumors (pooled from prospective trials) | 32% (95% CI 22% to 41%) | Urias et al.^1^ |
| RT + IO + TKI | Unresectable Hepatocellular Carcinoma (HCC) | 62.1% (18 of 29) | Yuan et al.^2^ |

Note: Representative grade ≥3 treatment-related AEs are summarized from the cited studies. Abbreviations: AE, adverse event; CI, confidence interval; HCC, hepatocellular carcinoma; ICI, immune checkpoint inhibitor; IO, immunotherapy; PD-1, programmed death-1; PD-L1, programmed death-ligand 1; RT, radiotherapy; SBRT, stereotactic body radiotherapy; TKI, tyrosine kinase inhibitor.

1.	E. Urias, J. Lee, C. R. Weil, et al., “Combined immune checkpoint inhibitors and ablative radiotherapy in metastatic cancers: a meta-analysis of prospective clinical trials,” BMJ Oncology 4 (2025): e000732

2.	Y. Yuan, Y. Chen, C. Huang, et al., “Efficacy and safety of radiotherapy combined with immunotherapy and targeted therapy versus immunotherapy plus targeted therapy alone in unresectable hepatocellular carcinoma: a retrospective study,” Frontiers in Oncology 15 (2025): 1643304
